# Livestock vaccination programme participation among smallholder farmers on the outskirts of National Parks and Tiger Reserves in the Indian states of Madhya Pradesh and Assam

**DOI:** 10.1371/journal.pone.0256684

**Published:** 2021-08-27

**Authors:** Andy Hopker, Naveen Pandey, Rosie Bartholomew, Abigail Blanton, Sophie Hopker, Aniruddha Dhamorikar, Jadumoni Goswami, Rebecca Marsland, Prakash Metha, Neil Sargison

**Affiliations:** 1 Royal (Dick) School of Veterinary Studies, Easter Bush Veterinary Centre, University of Edinburgh, Roslin, Midlothian, Scotland, United Kingdom; 2 The Corbett Foundation, Kaziranga Office, District Golaghat, Assam, India; 3 School of Social and Political Sciences, University of Edinburgh, George Square, Edinburgh, Scotland; Ashoka Trust for Research in Ecology and the Environment, INDIA

## Abstract

Effective livestock vaccination has the potential to raise prosperity and food security for the rural poor in low and middle income countries. To understand factors affecting access to vaccination services, and guide future policy, smallholder farmers in three locations in India were questioned about vaccination of their cattle and buffalo, with particular reference to foot and mouth disease (FMD), haemorrhagic septicaemia (HS) and blackquarter (BQ). In the three regions 51%, 50%, and 31% of respondents reported vaccinating their livestock; well below any threshold for effective population level disease control. However, within the third region, 65% of respondents in villages immediately surrounding the Kaziranga National Park reported vaccinating their cattle. The majority of respondents in all three regions were aware of FMD and HS, awareness of BQ was high in the Kanha and Bandhavgarh regions, but much lower in the Kaziranga region. The majority of respondents had positive attitudes to vaccination; understood vaccination protected their animals from specific diseases; and wished to immunise their livestock. There was no significant association between the age or gender of respondent and the immunisation of their livestock. Common barriers to immunisation were: negative attitudes to vaccination; lack of awareness of date and time of vaccination events; and difficulty presenting animals. Poor access to vaccination services was significantly associated with not vaccinating livestock. Fear of adverse reactions to vaccines was not significantly associated with not vaccinating livestock. Respondents who reported that vets or animal health workers (AHWs) were their main source of animal health knowledge were significantly more likely to have immunised their livestock in the last twelve months. Participants cited poor communication from vaccinators as problematic, both in publicising immunisation programmes, and explaining the purpose of vaccination. Where vaccinations were provided free of charge, farmers commonly displayed passive attitudes to accessing vaccination services, awaiting organised “immunisation drives” rather than seeking vaccination themselves. Based on these findings the following recommendations are made to improve participation and effectiveness of immunisation programmes. Programmes should be planned to integrate with annual cycles of: disease risk, agricultural activity, seasonal climate, social calendar of villages; and maximise efficiency for vaccinators. Dates and times of immunisation in each village must be well publicised, as respondents frequently reported missing the vaccinators. Relevant farmer education should precede immunisation programmes to mitigate against poor knowledge or negative attitudes. Immunisation drives must properly engage beneficiaries, particularly ensuring that services are accessible to female livestock keepers, and sharing some responsibilities with local farmers. Payment of a small monetary contribution by animal keepers could be considered to encourage responsibility for disease prevention, making vaccination an active process by farmers.

## 1. Introduction

Better efficiency of livestock keeping as part of mixed smallholder enterprises in low and middle income countries (LMICs) has the potential to improve resource utilisation, profitability, childhood nutrition, and empowerment of rural women [1–4]. Infectious diseases are a major cause of losses [5], and the effect on smallholder households is magnified due to reduction in milk for childhood nutrition; lost cultivation days by draught animals; reduction in manure for agricultural fertiliser; time and cost of nursing sick animals; expense and difficulty of sourcing replacement animals; and emotional harm suffered by households due to the death of animals, which may be highly regarded as part of the household [6]. Inefficiency in production, in part due to livestock disease, results in increased greenhouse gas emissions contributing to the climate crisis, which disproportionately affects the rural poor [5].

Smallholder farmers in LMICs are particularly at risk from infectious livestock disease plagues due to close proximity between animals from neighbouring holdings; the use of shared grazing lands and water resources; crowded livestock markets; limited access to livestock vaccinations; and absence of biosecurity measures. Vaccination of livestock has the potential to reduce these losses by reducing the frequency and severity of disease outbreaks. Barriers exist to farmers’ already limited access to livestock vaccinations, and additional socio- economic factors influence their decisions on accessing those vaccine services that are available [4, 7].

Approximately 66% of the population of India is classified as rural, about 892 million people, the great majority of whom are engaged in agriculture [8]. Most agricultural units are mixed cultivation and livestock smallholder enterprises; the average holding comprises just 1.08 hectares, a decrease of 1.2 hectares since 1970 [9]. India faces the challenge of feeding 18% of global population using 11% of cultivatable land [10]. Livestock play a key role in the efficiency and profitability of smallholder farming systems, producing valuable protein from marginal natural resources and agricultural residues, and providing accessible cash income for their keepers. Three disease plagues commonly affect cattle and buffalo in rural India: foot and mouth disease (FMD); haemorrhagic septicaemia (HS); and black quarter (BQ) [11]. Timely and effective vaccination can reduce suffering, improve profitability, be efficient in terms of animal keeper and animal health worker (AHW) time, and reduce reliance on prophylactic and therapeutic use of veterinary medicines [12].

Foot and mouth disease (FMD) is a highly infectious, production limiting, vesicular disease of ruminants which is endemic in India. Lameness and anorexia reduce productivity, and draught animals may be unable to work for an extended period, further impacting on smallholder productivity by delaying cultivation activities. Morbidity rates are high with FMD infection, but fortunately mortality rates are usually low when endemically infected populations of native breed animals are infected with local FMD serotypes [13], serotypes O, Asia 1 and A being prevalent in India [14]. In 2011, the estimated cost of FMD to the Indian economy was US$ 2.6 billion [15]. Outbreaks of FMD occur year-round [16], but are most common in the post- monsoon season [14]. It is a stated goal of the Indian Government to limit the spread and impact of FMD through the FMD-CP (Control Programme), vaccinating all cattle and buffalo twice annually, using a single primary injection followed by six monthly boosters [17, 18]. Vaccines are typically of Indian manufacture, and administered by veterinary clinicians or AHWs. Unfortunately uptake of vaccination has been poor in certain areas. Assam and Madhya Pradesh are the states with the lowest and third lowest levels of vaccine induced herd immunity in terms of antibody levels in the cattle population, and this has been related to increased FMD outbreaks in these states [17]. Where twice yearly vaccination is practiced there has been a reduction in cases of FMD [14, 17]. Vaccine coverage of 80% or greater can be considered good for prevention of disease transmission in a population through herd immunity, and below 50% as poor [17]. Vaccine acquired herd immunity is more important for the population than the individual [17], and is the most practical method to reduce disease transmission where biosecurity controls are weak.

Haemorrhagic septicaemia (HS) is an acute bacterial disease affecting buffalo and cattle caused in India by serotypes of *Pasteurella multicoda type B*, characterised by respiratory distress and inflammation of the larynx and trachea. HS is usually fatal, death typically occurring 24–48 hours after clinical signs are first noticed, though can present as sudden death [19, 20]. The annual financial impact of HS in India is estimated at US$ 683 million. Outbreaks are more sporadic than FMD, HS occurs most commonly during the wet season [21], or immediately after the monsoon [19] as organism survival and transmission is greater in moist or humid conditions [20]. Successful treatment of HS is difficult due to the peracute nature of the disease, compounded by poor access to quality antibiotic drugs and increasing antimicrobial resistance [22]. Vaccination is the only practical method of protecting animals from HS in endemic regions [20]. Effective vaccines against HS are available in India, however, these generally do not provide 12 months of protection, and due to this outbreaks still occur [23].

Blackquarter (BQ), known as Blackleg in the UK, is caused by the spore-forming, soil bacteria, *Clostridium chauvoei* [24]. Typically, animals ingest spores while grazing, which remain dormant in muscles, until bruising from trauma creates a suitable anaerobic environment. Outbreaks of BQ are more common following earthworks, or associated with heavy rain or flooding [25]. BQ is characterised by myonecrosis, generalised toxaemia, and death [24]. Dark red rancid-smelling fluid sometimes exudes through the skin overlaying affected muscles, which are swollen and painful. Occasionally cattle may recover from BQ with aggressive treatment following an extended period of illness and exudation [26]. Outbreaks of BQ on the Indian subcontinent are most common after the monsoon, financial losses are primarily due to mortality [27], the annual cost of BQ in India has not been estimated. Spores persist in soil for many years, and as treatment is usually ineffective, vaccination is the only practical means of disease control [28]. Vaccination against BQ should be administered as two primary injections given one month apart, followed by an annual booster. Immunisation against BQ is generally effective, providing one year of protection [29], disease among vaccinated animals is assumed to result from problems with vaccine handling and administration [28].

Domestic animals at the human-livestock-wildlife interface are a key threat to wild populations through cross- species disease spread, and wild populations also act as transmitters of infection to domestic species [30]. Little is known about FMD in wild animals, and most information is extrapolated from domestic species. With respect to FMD, outside sub- Saharan Africa disease transmission is primarily from domestic species to wild populations, and disease in wild animals can be severe and acute [31]. A study of an FMD and HS outbreak in the Bannerghatta biological park, Karnataka, India, which rapidly followed an outbreak among domestic livestock in neighbouring farms reported the discovery of the affected carcasses of two Indian Guars, seven nilgias, and 49 other deer of three species, though the true extent of the outbreak is unknown. Identification of the virus revealed FMD type O of a lineage prevalent in local livestock, and these dead animals were co- infected with Pasteruella multocida type B (HS) of a serotype commonly found in Indian domestic ruminants [32] Close proximity of large numbers of susceptible animals is a prerequisite for disease outbreaks. Disease control by vaccination of livestock therefore potentially benefits wildlife [33]. A study in the Kruger National Park, South Africa, modelling the spread of FMD from wild African buffalo to domestic cattle predicted that 75% of cattle would have to be effectively immunised, as well as a reduction in the number of cattle/ buffalo contacts, would be required to control FMD in cattle South Africa [34]. An Australian study modelling FMD transmission between wild pigs and domestic cattle similarly reported that control efforts would have to focus on both species [35]. The sporadic nature of cases of BQ, long persistence of infective spores in soil, the occurrence of clinical disease a highly variable time after spore ingestion, and rapid post mortem purification of affected carcasses make it difficult to accurately quantify losses due to this disease in any circumstances where veterinary public health surveillance is challenging to undertake. A study of 360 farmers in the state of Uttarakhand, Uttar Pradesh, Punjab and Haryana found significant regional variations in attitudes to livestock vaccination. A majority of farmers in all regions believed vaccination to be relevant, however only in Punjab and Haryana did a majority also believe livestock vaccination to be profitable. The authors attribute the main source of difference in attitudes to immunisation to be level of commercialisation, with commercial farmers holding more positive views of livestock vaccination than smallholder farmers [36]. A study of 601, mostly urban, poor livestock keepers in Tamil Nadu found understanding of the causality of livestock disease to be the most important determining factor in the uptake of immunisation [37]. Human public health studies have closely examined reasons for poor vaccine coverage among children in India. These include lack of awareness of the need for vaccination; poor knowledge of immunisation schedules; busy daily schedules; travel or migration; fear of adverse effects; lack of health services; and distance to the place of vaccination [38]. Mothers with better health literacy were more likely to access vaccination services for their children than those assessed as less health literate [39], and provision of relevant health education to mothers prior to the implementation of vaccination programmes has been shown to improve vaccination rates [40]. Long term sustainability of small holder livestock vaccination programmes requires that animal keepers share the cost of vaccination. A study of Newcastle Disease poultry keepers in Tanzania found that previous vaccination was the most important factor in willingness to pay for immunisation, and that on–farm income was a sufficient driver for affordability of immunisation [41]. Investigation of willingness to pay for FMD vaccination among livestock farmers in Ethiopia found that overall 59% were willing to pay, and this increased to 75% among market orientated livestock only farmers. Increased knowledge of vaccines and FMD were significantly associated with increased willingness to pay [42].

FMD, HS and BQ can all be mitigated by vaccination, individual vaccines and a trivalent vaccine protecting against all three diseases are available in India, the trivalent vaccine stimulating a similar immunological response to single pathogen vaccinations [42]. Available vaccines for FMD and HS do not provide 12-months protection, a UK study found adequate protection to be present six months after vaccination with a single injection [42]. Therefore planned immunisation programmes must vaccinate animals prior to the expected period of greatest risk [43]. However, as the seasonal occurrence of these diseases differ, it is difficult to achieve control of all three diseases using once yearly vaccination with trivalent vaccine. This difficulty is compounded by administering a single injection as the initial immunisation, which could result in suboptimal level and duration of immunity [44–47].

**Fig 1:**
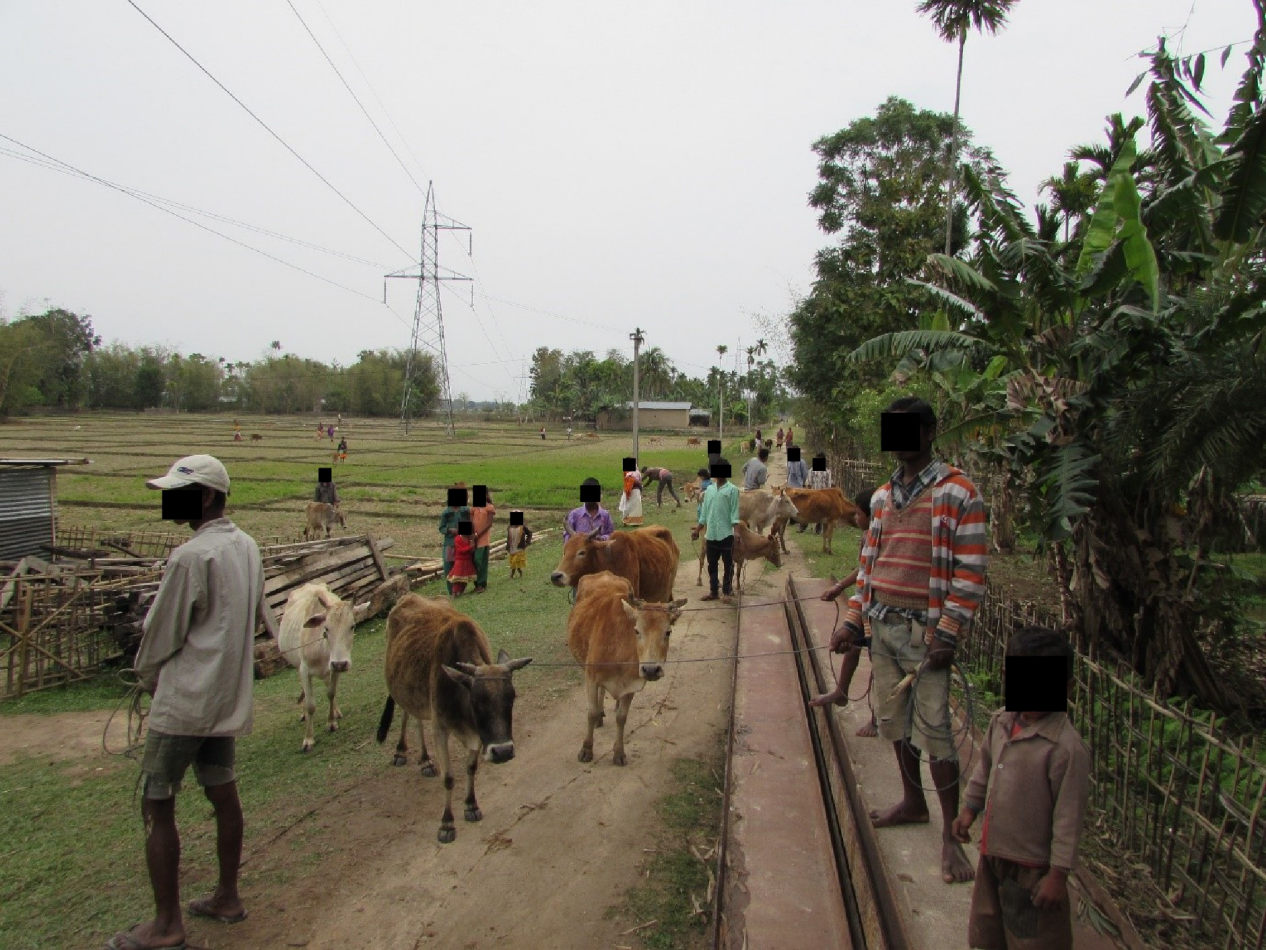
Cattle being gathered for foot and mouth vaccination. Vaccine drives may involve owners bringing their animals to a single site. These programmes are efficient in terms of animal health workers’ time. However, some animals may be missed due to: long walking distances involved for people and their animals to reach the vaccination or treatment sites; difficulties in catching and restraining animals; or animal keepers being otherwise occupied at the time. It is noteworthy that small ruminants and pigs are not included in this foot and mouth vaccination drive. Furthermore, bringing animals together in this way could increase the risk of virus transmission, and spread of other production limiting infectious diseases.

**Fig 2:**
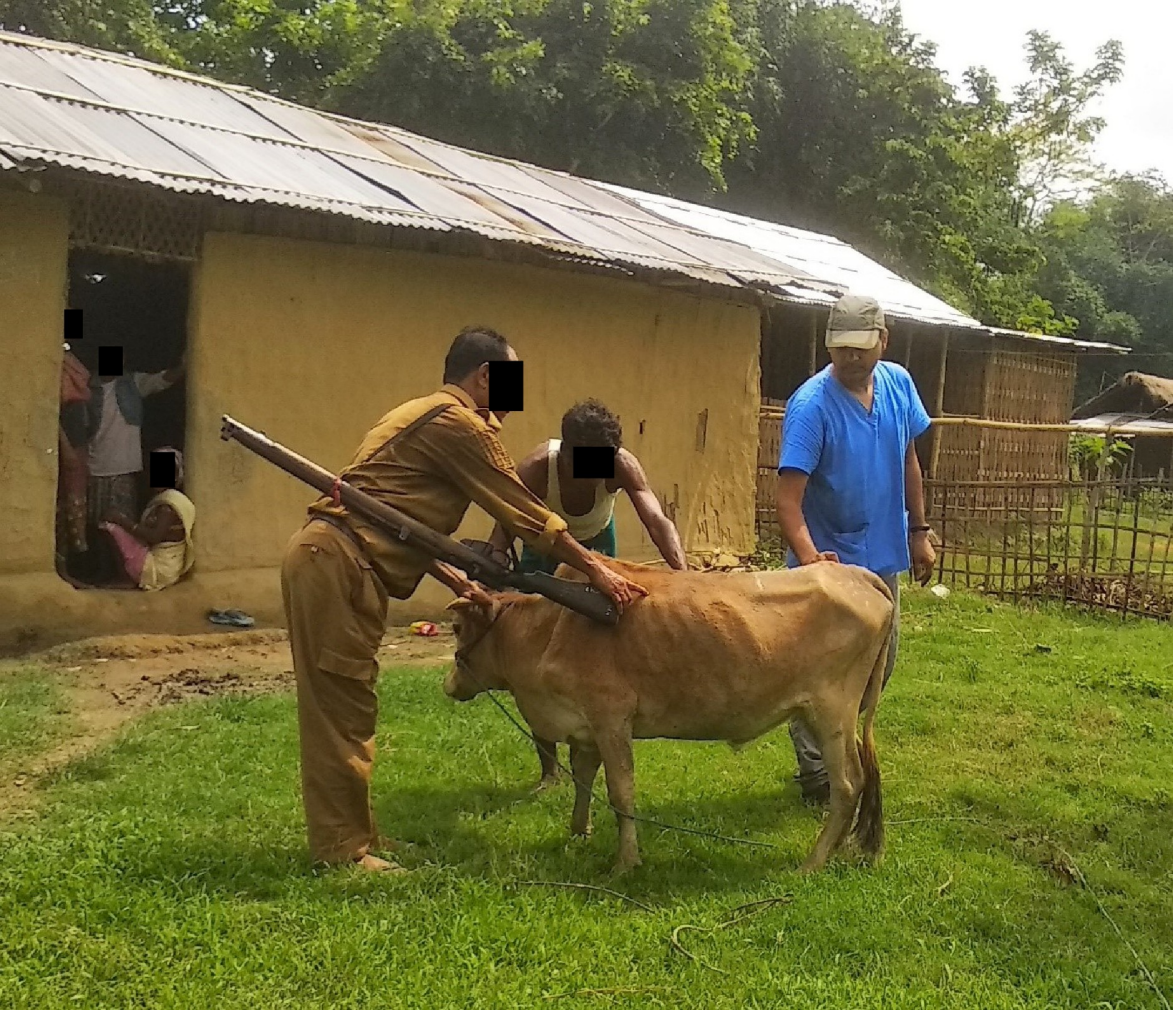
Vaccination team (Forest Dept/ TCF) visiting cattle in their own accommodation for immunisation near the Kaziranga National Park. Vaccination teams visiting every household in a village improves coverage as fewer animals are missed. Owners do not have the problem of restraining their animals in an open area; mixing of animals, which may spread disease, is reduced; and this approach facilitates dialogue between vaccinators and animal keepers.

**Fig 3:**
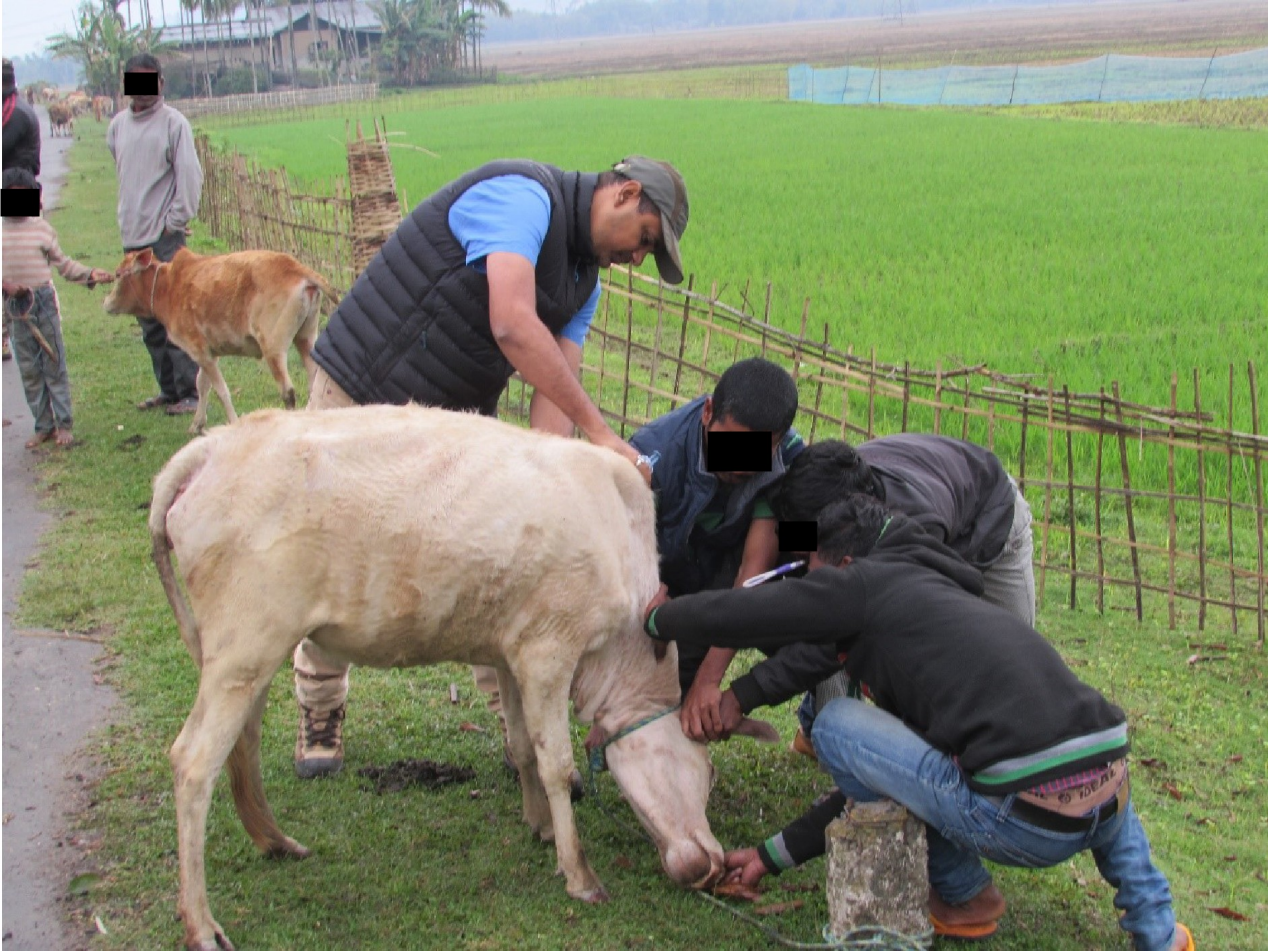
Cattle presented for vaccination at the roadside in front of the keepers house on the fringe of Kaziranga National Park, Assam. Presentation of livestock in front of the keepers home is an effective compromise, allowing vaccination teams to work efficiently. This is an example of the benefit of community engagement in the planning and undertaking of immunisation programmes.

Vaccination programmes for village cattle and buffalo close to forest reserves in Madhya Pradesh and Assam are provided by the Forest Department and Non- Governmental Organisations (NGOs), such as The Corbett Foundation (TCF), with the aim of protecting livestock and reducing the spread of infection to wild species within the National Parks and Tiger Reserves [48]. Livestock immunisation programmes may be undertaken either by gathering animals in one place or with the vaccination teams working door to door to visit the animals at home (Figs 1–3). Vaccination programmes which are undertaken prospectively as part of planned animal management programmes achieve better herd level coverage [48] than *ad-hoc* initiatives in response to disease outbreaks, and a planned approach allows livestock to develop immunity prior to the predicted period of maximum disease risk. It is often challenging to achieve a level of vaccine coverage that affords adequate control of disease spread through herd immunity. The aim of this work is to better understand reasons why livestock keepers do, or do not, vaccinate their animals, and the factors influencing these decisions. This will aid in the development of pragmatic recommendations to sustainably improve livestock vaccine coverage, including the use of domestic livestock vaccination programmes to help conservation efforts for wild species.

## 2. Materials and methods

### 2.1 Study area

**Fig 4:**
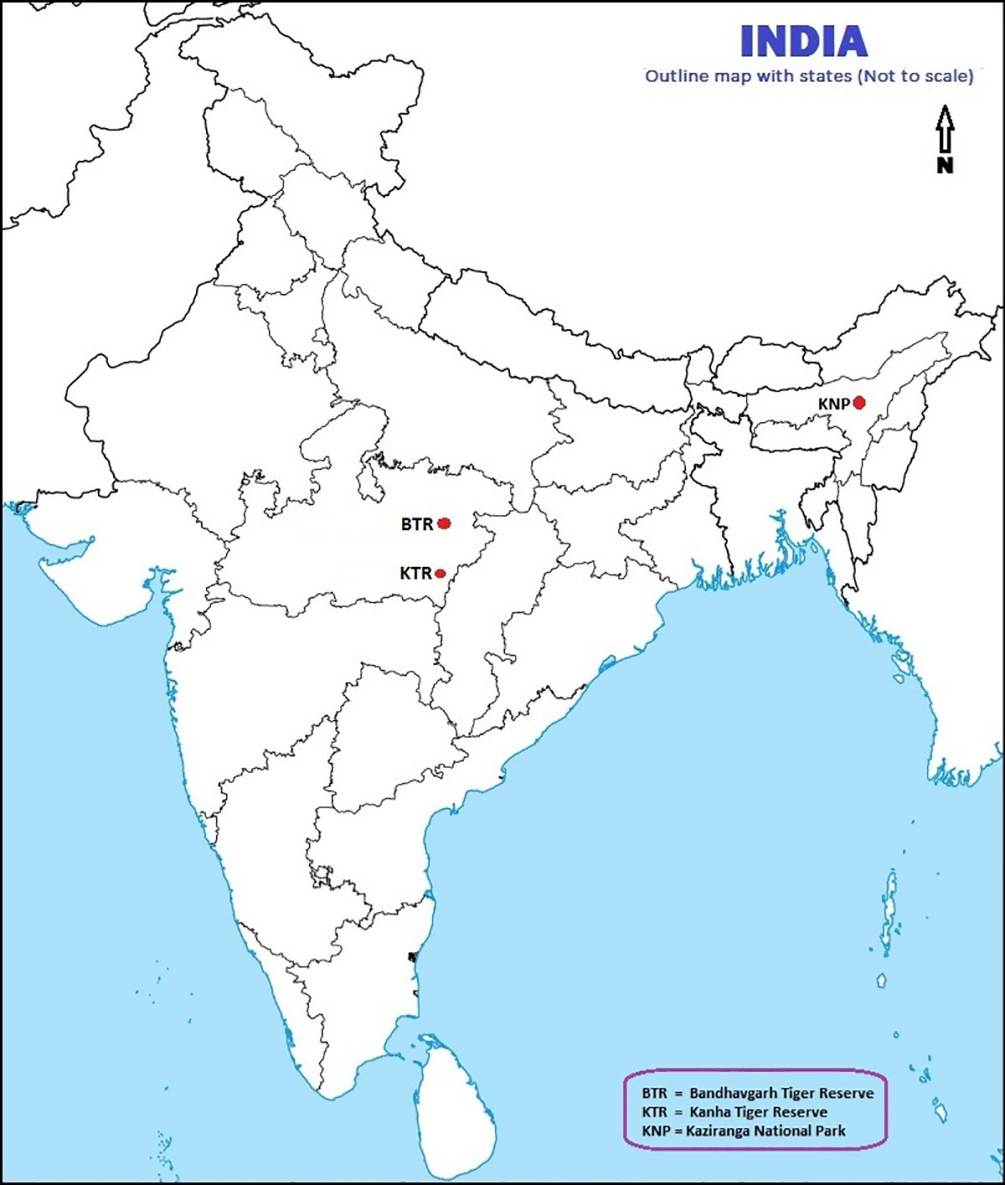
Map of India indicating the locations of The Kanha Tiger Reserve (KTR, Bandhavgarh Tiger Reserve (BTR), and Kaziranga National Park (KNP).

The Kanha Tiger Reserve (KTR) (Fig 4) in Madhya Pradesh state in the Central Indian Highlands has a 917 sq km Core zone and a 1,134 sq km Buffer zone. Entry into the Core zone is strictly controlled using a permit system. The Buffer zone contains 183 villages, home to around 129,300 humans in 2015 [49]; agriculture is the main form of income in these settlements. Farms are mixed smallholdings, typically less than two acres in size. Entry into the Buffer zone is monitored by the Forest Department. Ruminant livestock represent an important source of productive income and draft power for inhabitants of the Buffer zone.

The Bandhavgarh Tiger Reserve (BTR) is also located in Madhya Pradesh, in the Vindhya Hills (Fig 4). The BTR encompasses a core area of 716 sq km and a buffer area of 820 sq km. Entry system is similar to the KTR, except that ten traditional villages are present within the Core zone as well as 152 villages in the Buffer zone, the human population of this area in 2017 was estimated to be 97,556 individuals [50]. Livestock usage is also similar to KTR.

The Kaziranga National Park (KNP) in Assam lies along the Brahmaputra River spanning parts of Golaghat and Nagaon districts (Fig 4). The Brahmaputra river borders the parkland to the North and East, the Mora Diphlu river provides the southern border. The KNP includes 378 sq km of land and part of the adjacent section of the Brahmaputra River. Entry into the KNP is strictly forbidden, other than ticketed tourist entry, or on Forest Department business. The KNP has no Buffer zone, there are 74 nearby villages considered of importance to the management of the National Park. During 2014 the human population of this area was calculated to consist of 13,663 families, with a total population of 66519 people [51] Agricultural activities are undertaken right up to the park boundary. There are 46 villages of particular proximity which have been the beneficiaries of immunisation initiatives by the Forest Department and NGOs in 2018 and 2019 [51].

All three regions are areas of activity of The Corbett Foundation (TCF), including livestock vaccination programmes designed to protect wild species within the National Parks and Tiger Reserves, to assist village inhabitants with sustainable livelihoods through efficient farming, and improve the welfare of domestic livestock through reduction in suffering due to endemic disease.

### 2.2 Data collection

Information about access to vaccination services, attitudes to vaccination and the effects of disease plagues were collected through a combination of structured survey interviews and in- depth open interviews.

Survey interviews were carried out in villages around the KTR, the BTR, and the KNP. Interviews were carried out at 25 villages around the KTR between 12^th^ July and 1^st^ August 2016, contacting 143 respondents; at five villages around the BTR between 20^th^ and 24^th^ June 2017, with 22 respondents; and at 29 villages around the KNP between the 1^st^ and 10^th^ April 2019, contacting 140 respondents. In each location interviews were carried out by a local TCF community worker and a veterinary student or clinician. Interviews were undertaken in the local languages, Hindi in the KTR and BTR, and Assamese in the KNP region.

Structured survey interviews used closed questions after which participants were asked to explain their responses. Questions were designed to discover whether or not farmers accessed cattle vaccination programmes and the reasons behind their decisions; barriers to accessing vaccination; levels of disease awareness and knowledge of cattle vaccination; perceived impacts of vaccinatable diseases; perceptions of the effectiveness of vaccination; and the effects of charging for vaccination. The age and gender of respondents, and the number of cattle and buffalo owned was recorded. The survey questionnaires differ slightly between the Kanha/ Bandhavgarh part of the study and the Kaziranga part. This is because these questionnaires were developed by veterinary students, with guidance, to form part of their research projects. While the supervising authors ensured consistency across the study it was also important to allow the students a degree of autonomy to develop their own survey tools. Full details of the survey questionnaires are shown in S1 File. Survey interviews typically took 12–20 minutes per respondent.

Village locations were chosen by local TCF community workers with the hope of being representative of the region, including local ecological features, and to include farmers from Scheduled Tribes and Scheduled Castes, as well as farmers from General Castes. In each study area a convenience sample of village inhabitants was used. Dwellings within each village were chosen at random and reached via a door-to-door approach. One respondent was interviewed from each household, the adult householder who identified themselves as the main livestock carer. No other recruitment criteria were applied, and respondents were not asked if they identified as any caste or tribe.

In-depth interviews were carried out around the KNP with 18 households, between 13^th^ and 25^th^ of March 2017 in three villages close to the KNP boundary. These villages are among those benefiting from Forest Department immunisation programmes. Potential respondents were contacted through word of mouth, and a purposeful sample selected by TCF community workers to try to represent the variety of village inhabitants, including a balance of gender, age, social status, relative wealth, level of education, and additional livelihoods. This work formed part of a smallholder farmer education programme in the region. Interviews were carried out at the participants’ homes, employed open questions with follow up discussions, and typically lasted two to three hours. Interview discussions were wide ranging, incorporating animal health and husbandry, planned preventative medicine, access to veterinary services, household livelihood strategies, animals kept and their uses. An *aide-mémoire* was used to ensure that certain areas were consistently covered. Only information relevant to livestock vaccination is included in this manuscript. Full details of the interview process are shown in S2 File. Interviews were conducted in Assamese by a local Assamese community worker already known to the participants, and a UK veterinary surgeon. Participants were encouraged to freely discuss any subject of importance or interest to them. All participants were volunteers, introduced to the project by a friend or relative, no household declined to take part in the study.

This work was planned, undertaken and reported in accordance with the COREQ guidelines for qualitative research [52]. The aims and scope of the study were explained to all participants, and informed verbal consent recorded. The use of signed consent forms was not appropriate due to local mistrust of signed documents and varying levels of literacy. Respondents were informed that participation was anonymous, and that they were free to withdraw from the study at any time. Interview notes were transcribed and NVIVO 11 (QSR International Ltd) was used to build a node structure, to which responses were coded for analysis. Where appropriate statistical testing was performed using chi- square tests via Minitab (Minitab 19, Minitab LLC). TCF maintains excellent relations with the local Forest Department divisions, however as this work was undertaken in the fringe villages outside the National Park areas no specific permissions were required.

### 2.3 Ethical approval

Ethical oversight and approval was provided by the Edinburgh University Human Ethics in Research Committee (HERC 47–17).

## 3. Results

### 3.1 Survey interviews around the KTR, BTR and KNP

In the area surrounding the KTR 125 male and 18 female livestock keepers, aged 19 to 72 years (mean 38) were interviewed. In the BTR area 19 male and 3 female livestock keepers were interviewed, aged 24 to 66 years (mean 45). In the area surrounding the KNP 62 male and 78 female livestock keepers (mean age 43 years) were interviewed. In the KNP area 22 respondents lived in villages bordering the National Park which had been the beneficiaries of the 2018–2019 immunisation drive. The mean (median; range) size of a household’s combined herd of cattle and buffalo of all ages, was 4 (4; 1 to 22), 12 (11; 5–22) and 5 (4; 1–20) animals in the KTR, BTR and KNP areas respectively. Full details of the results of the survey interviews can be found in the S3–S5 Files.

Knowledge of livestock keepers awareness of specific diseases, their perceived importance and prevalence, is essential if future immunisation programmes are to be designed to maximise saturation through engagement of animal keepers. Livestock keepers were asked if they were aware of each of the three diseases (FMD, HS, and BQ), if their animals had suffered from each disease, and which of the three diseases they considered of the greatest importance (or threat) to them, when considering all factors including: frequency of occurrence, rate of spread, mortality, cost and efficacy of treatment, financial loss, and emotional impact. In all three regions, FMD was the disease most commonly reported to have affected the participants’ cattle and buffalo (Table 1). FMD was the disease that the greatest number of participants were aware of in the KTR and KNP groups. In the BTR group awareness of BQ was greatest. The KTR group considered HS the most important disease, and HS was reported to be more common by the KTR group than the others. BQ was reported as more prevalent by the BTR group than the other groups, and BQ was considered most important by the BTR group. The KNP group considered FMD to be the most important disease. Respondents around the KNP perceived HS and BQ to be less prevalent than respondents in the other regions.

**Table 1:** Disease awareness and vaccine usage.

		Kanha (KTR)	Bandhavgarh (BTR)	Kaziranga (KNP)
Number of respondents		143	22	140
% Female respondents		13%	14%	56%
“I have heard of FMD”		**127 (88%)**	21 (95%)	**140 (100%)**
“My animals affected by FMD”		**60 (42%)**	**19 (86%)**	**118 (86%)**
“I have heard of HS”		108 (76%)	15 (68%)	107 (76%)
“My animals affected by HS”		37 (26%)	11 (50%)	33 (24%)
“I have heard of BQ”		114 (80%)	**22 (100%)**	6 (4%)
“My animals affected by BQ”		35 (24%)	13 (59%)	1 (0.7%)
“In my opinion this is the most important cattle disease”	FMD		34 (24%)	5 (23%)	**125 (90%)**
	HS		**60 (42%)**	2 (9%)	4 (3%)
	BQ		35 (24%)	**9 (41%)**	1 (0.7%)
	More than one		3	6 (27%)	2 (1.5%)
	Other disease		0	0	4 (3%)
	Don’t know		11	0	4 (3%)
“I vaccinated my cattle or buffalo last year”		**51%** (73/ 143)	**50%** (11/ 22)	**31%** (44/ 140)
“I vaccinate my cattle every year”		81% (117/143)	55% (12/22)	91%* (40/44)
“Vaccination helps to protect from disease”		71% (101/ 143)	68% (15/ 22)	61% *(27/ 44)
Would you be willing to pay for vaccine?		0%**	0% (0/22)	95% *(42/ 44)
Has number and severity of disease outbreaks increased or decreased since vaccination started?	Decreased:	105 (83%)	19 (86%)	118 (84%)
	Increased:	15 (12%)	3 (14%)	1 (0.7%)
	Don’t know:	7 (3%)		21 (15%)
		n = 127	n = 22	n = 140
			
Can purchased livestock introduce disease?	Yes:	107 (83%)	8 (36%)	101 (72%)
	No:	19	10	3
	Don’t know	2	4	36
		n = 128		

**Note:** Bold type highlights priority findings, including diseases with highest perceived importance or awareness among respondents.

*In the KNP only respondents who reported vaccinating their animals (n = 44) were asked why they did so, how often, and if they were willing to pay for vaccination.

**In the KTR the investigation team were unable explain the concept of paying for vaccine to the participants, who typically responded “*But I don’t pay for vaccine*.”

Population saturation by an immunisation programme is important not only for the protection of individual animals, but also for protecting populations of both domestic and wild animals by reducing onward transmission of disease. Among the KTR and BTR groups 51% and 50% of respondents reported that they had vaccinated their animals this year. In these regions there had been a recent vaccination drive by TCF. Around the KNP group 31% of respondents reported having vaccinated their livestock in the previous year (2018–2019). Recent vaccination drives in this region by the Forest Department and NGOs had concentrated on protecting the National Park by ring vaccination, working in villages on the fringe of National Park, and 65% of respondents (13/22) from these fringe villages reported vaccinating their livestock in the previous 12 months (2018 immunisation programme beneficiaries). Cattle and buffalo in all three locations were typically vaccinated with a single injection of trivalent vaccine (Raksha Triovac^TM^, Indian Immunologicals Ltd) offering protection against FMD, HS and BQ.

Understanding the effect of the monetary cost of vaccination as a potential barrier to immunisation, is important in the design of future programmes. When asked if they would pay for livestock immunisation, no respondent in the KTR or BTR groups said that they would be prepared to pay, and in the KTR the interviewers struggled to explain the concept of paying for vaccination services to farmers, a typical response was *“But I don’t pay for vaccination*.*”*. The perception of vaccination as a service which is provided rather than sought has important implications for the development of sustainable future immunisation programmes, where livestock keepers share the cost of vaccinations. In the KNP group 95% of respondents who had had their livestock vaccinated in the last 12 months said that they would be prepared to pay for livestock vaccination. Respondents in the KNP group who had not had their animals vaccinated in the last 12 months were not asked if they were willing to pay or not, in this region these questions were only posed to farmers who reported immunising their livestock. However seven non- vaccinating respondents stated independently that they would be willing to pay.

“*It costs about Rs 40 to vaccinate an animal… If someone organised a yearly vaccination program it would be helpful for us*. *From the village they need money and we are ready to pay*. *They would be responsible for buying and administering vaccines themselves*. *From the government it is free*.*”*

                KNP participant 129, female, 31–40 years.

Recognition of potential sources of infection is a prerequisite for reducing disease challenge by implementing basic sustainable biosecurity procedures. It is also a key factor in properly enfranchising farmers in immunisation programmes. Livestock keepers were asked about potential sources of disease risk to their animals. Purchased livestock brought from outside the village were identified as a risk by 83% of respondents in the KTR group, 36% in the BTR group, and 72% in the KNP group (Table 1). Among the KNP group 46 respondents stated that they believed disease spread between animals from different households occurred while the animals were at shared or rough grazing. Within the KNP group, 15 respondents identified the seasonal flood as having a role in disease transmission.

Understanding demographic trends in accessing vaccination services could help target pre- vaccination education activities intended to maximise uptake of immunisation. No trend linked age of respondents with likelihood of vaccinating their animals in any of the three regions (Chi square test [4, n = 299] likelihood ratio 7.29, P = 0.121, six respondents who did not state their age were excluded) (Table 2). The effect of gender was inspected only in the KNP group, due to the low proportion of female respondents in the KTR and BTR groups. Male respondents in the KNP group had vaccinated their animals more frequently than female respondents (Table 3), however this difference was not significant (odds ratio = 1.60, chi square test [1, n = 140} likelihood ratio 1.653, P = 0.198)and this finding should be considered with reference to other factors affecting women’s access to vaccination services. These include the timing of immunisation drives in the mornings when women are often occupied with other tasks, such as household routines, including getting children ready for school, domestic duties, care of infants and elderly relatives. The difficulty of restraining cattle manually with extremely limited handling facilities. Reduced access to farmer education and extension services, all of which may ultimately disadvantage women in accessing livestock immunisation services.

**Table 2:** Distribution of animal keeper age compared to vaccination of livestock.

Region	Participant age group (years)	Vaccinated animals in the last year		Percentage of participants in age group who vaccinated their cattle last year
		Yes	No	
**Kanha(KTR)**	</ = 20	2	5	
	21–30	29	20	59%
	31–40	16	15	52%
	41–50	11	18	38%
	>50	13	12	52%
	Age not stated	2		100%
**Bandhavgarh(BTR)**	</ = 20	0	0	
	21–30	2	3	40%
	31–40	3	2	60%
	41–50	1	3	25%
	>50	5	3	63%
**Kaziranga (KNP)**	</ = 20	0	1	0
	21–30	5	8	38%
	31–40	12	26	43%
	41–50	15	33	31%
	>50	11	25	31%
	Age not stated	1	3	25%

**Table 3:** Animal keeper gender and livestock vaccination in Kaziranga.

Kaziranga (KNP) only	Male respondent		Female respondent	
	yes	no	yes	no
Vaccinated last 12 months? (n = 140)	23 (37%)	39 (63%)	21 (27%)	57 (73%)
Willing to pay for vaccine? (n = 44)	23 (100%)	0	20 (95%)	1 (5%)

**Note:** Percentages in brackets show proportion of respondents by gender vaccinating/ not vaccinating, or willing/ unwilling to pay for vaccination.

All respondents who vaccinated their animals during the previous year in the BTR and KNP groups stated disease prevention as the reason (Table 4), as did 70 of the 73 respondents who vaccinated in the KTR group. The other three respondents in the KTR did not know why their animals had been injected. In addition to disease prevention one respondent in the KTR mentioned “*preventing disease spread”* and another “*preventing outbreaks occurring during monsoon time*”. Among respondents from the KNP three farmers expanded on this to say “*vaccination of sufficient animals prevents the spread of disease*”, two respondents stated “*the timing of the vaccine administration was important*” and two said *“vaccinating my animals gives me a feeling of security*.”

**Table 4:** Reason for vaccinating/ not vaccinating livestock in the last 12 months.

Reason for vaccinating/ not vaccinating livestock this year	Kanha (KTR)			Bandhavgarh (BTR)			Kaziranga (KNP)		
	Participant vaccinated livestock last year?		Total participants citing	Participant vaccinated livestock last year?		Total participants citing	Participant vaccinated livestock last year?		Total participants citing
	yes	no		yes	no		yes	no	
Disease prevention	70	16	86	11	4	15	44	-	44
“Vaccination team did not come here last year”	-	2	2	-	-	-	-	28	28
“Vaccination team never comes here”	-	1	1	-	-	-	-	39	39
“I was not informed / did not have time to get my animals”	-	-	-	-	2	2	-	8	8
“I was not available, vaccinators come for a short time only”	-	30	30	-	-	-	-	4	4
“Animals had gone to the fields”		1	1	-	-	-	-	2	2
“I could not restrain my animals”	-	-	-	-	2	2	-	-	-
“I haven’t vaccinated yet this year”	-	1	1	-	-	-	-	-	-
“Swelling at the injection site”	-	3	3	-	1	1	-	-	-
“Cow is pregnant”	-	1	1	-	-	-	-	6	6
“Fever can occur after vaccination”	-			-	1	1	-	-	-
“I have heard animals may die after vaccination”	-	-	-	-	1	1	-	-	-
“My animals are well, so I don’t need to vaccinate them”	-	-	-	-	-	-	-	7	7
“I am buying in new cattle—no need to vaccinate them”	-	1	1	-	-	-	-	-	-
“I don’t know”	3	13	17	-	-		-	-	-
No awareness of vaccination at all		1	1	-	-		-	2	2
**Total**	**73**	**70**	**143**	**11**	**11**	**22**	**44**	**96**	**140**

Among participants who did not vaccinate their animals in the KTR group, 31 respondents were not able to present their animals when the vaccination team were in the village. 13 KTR respondents did not know why they had not vaccinated last year. In the BTR group, two respondents were not able to restrain their animals for vaccination and two were not informed of the vaccination drive. In the KNP group, 67 respondents said that vaccination teams did not visit their village. Difficulty accessing vaccination services was the most important determinant in whether cattle were vaccinated (chi square test [1, n = 305] likelihood 183, P > 0.001).

“*We want to vaccinate*. *If the government takes initiative for vaccination we are always ready to vaccinate animals*, *but they never come here or give any kind of information*.*”*

                KNP participant 120, male, 41–50 years.

“*They don’t come to the south side of the highway*. *They came 13 years ago*, *but haven’t come since*.*”*

                KNP participant 14, female, 31–40 years.

Another twelve respondents in the KNP group said that vaccination teams had come, but they were not informed in time to be able to present their animals, or had difficulty keeping their animals restrained, particularly as overnight livestock accommodation is not spacious, and grazing the primary source of nutrition:

“*They announce (*the vaccination drive*) the day before*. *They come only on one day for few hours and I wasn’t here”*

                KNP participant 19, male, 41–50 years.

“*Sometimes they come late and we’ve already released our animals to graze*. *Proper timing not used*, *so it is very difficult to bring them (*our animals*) to the place to vaccinate*. *If they come late we can’t keep animals in the shed*, *so it is difficult”*

                KNP participant 97, female, 41–50 years.

A minority of respondents (KTR 34%; BTR 14%; KNP 9%; overall 21%) were concerned about adverse effects of vaccination, and another small group believed that healthy animals do not require vaccination (2.6%). A greater problem is access to vaccination services, sited by 31% of respondents as their reason for not vaccinating livestock, though there is considerable regional variation in this finding (Table 6).

Disease prevention was the most commonly cited benefit of vaccination, by 66%, 77% and 79% of respondents in the KTR, BTR and KNP groups respectively. Positive but vague responses included “keeps animals healthy” and the literal “yes, vaccination is beneficial” (Table 5). In the KTR group, five respondents also stated that it was financially beneficial to vaccinate livestock:

“*Last year treatment for kurra patta and chaboka cost (our household) Rs*. *1200”*

                KNP participant 69, female, 41–50 years.

**Table 5:** Respondents views on the benefits of vaccinating livestock.

Benefits of vaccinating livestock	Kanha (KTR)			Bandhavgarh (BTR)			Kaziranga (KNP)		
	Participant vaccinated livestock last year?		Total participants citing benefit of vaccination of livestock	Participant vaccinated livestock last year?		Total participants citing benefit of vaccination of livestock	Participant vaccinated livestock last year?		Total participants citing benefit of vaccination of livestock
	yes	no		yes	no		yes	no	
Disease prevention	52	42	94	10	7	17	42	69	111
Disease prevention and financial reasons*	5	0	5	-	-	-	-	-	-
“Keeps animals healthy” and “prevents all diseases”	1	1	2	-	-	-	2	15	17
“Vaccine cures disease”	-	-	-	-	-	-	0	1	1
“Yes, vaccination is beneficial”	1	0	1	-	-	-	-	-	-
“No, vaccination is not beneficial”	0	5	5	1	0	1	-	-	-
“I don’t know”	13	21	34	0	4	4	0	11	11
Did not answer question	1	1	2	-	-	-	-	-	-
**Total**	**73**	**70**	**143**	**11**	**11**	**22**	**44**	**96**	**140**

* **Note:** “Financial reasons” includes “saves money”, “maximise animal feed intake”, and “maximise working days (for draught animals)”

*Kurra patta* and *chaboka* are local Assamese terms indicating foot lesions, and oral lesions and anorexia, caused by FMD.

One respondent in the KTR group and one in the KNP group stated that vaccination helps to prevent disease spreading to wild species within the National Park/Tiger Reserve. It is reasonable to assume that more respondents were aware of this, as respondents in all locations stated that they did, or did not, receive free livestock vaccinations because of their proximity to (or distance from) the Park or Reserve.

Potentially serious misconceptions about livestock immunisations, including participants expressing a lack of knowledge, a negative attitude to vaccination, unrealistic expectations *(“vaccination prevents all disease”)*, or the belief that vaccination treats pre-existing disease accounted for 39% (49/143) of respondents in the KTR group, 23% (5/22) in the BTR group, and 9% (12/140) in the KNP group. The majority of participants who didn’t know the purpose of vaccination had not vaccinated their livestock in the previous 12 months, among respondents who said they did not know the purpose of vaccination, 62% in KTR and 100% in BTR and KNP had not immunised their livestock. Knowledge of which diseases their animals were protected against was variable among animal keepers who had vaccinated their animals. Of the 44 participants from the KNP group who reported vaccinating their animals during the previous year, 27 (61%) believed that their cattle had been vaccinated against FMD and 17 (39%) did not know what diseases their cattle had been vaccinated against. Respondents frequently cited poor communication from vaccination parties as the cause of this:

“*…they never say what disease*, *they just come and inject”*

                KNP Participant 17, male 31–40 years.

“*Someone asked a question about the vaccines but the doctor did not inform them*. *They were not interested in answering*.*”*

                KNP participant 134, female 31–40 years.

Measures to optimise uptake of livestock immunisation programmes need to address reasons why some farmers actively choose not to vaccinate, as well as factors which are a barrier preventing farmers who do wish to vaccinate their livestock from doing so. When respondents were asked if there were reasons not to vaccinate their livestock, the most common answer in all three locations was that there were no reasons not to vaccinate (KTR 34%; BTR 73%) (KNP 80% of respondents who had vaccinated their animals) (Table 6). Adverse reactions to the vaccine were cited by 34% (48/143) in the KTR group, 14% (3/22) in the BTR group, and 9% (12/140) in the KNP group. However, fear of adverse reactions had no statistical effect on whether or not animals were vaccinated (chi square test [1, n = 305] likelihood ratio = 2.515, P = 0.113). The most common adverse reaction mentioned by respondents was swelling at the injection site, other concerns included ‘death’, ‘weakness’ and ‘fever’. Some participants believed that pregnant animals should not be vaccinated. In the KTR group, 22% of respondents (32/143) said that they didn’t know if there were reasons not to vaccinate livestock, or declined to answer the question.

**Table 6:** Participants perceptions of reasons not to vaccinate livestock.

Are there reasons not to vaccinate livestock?		Kanha (KTR)			Bandhavgarh (BTR)			Kaziranga (KNP)		
		Participant vaccinated livestock last year?		Total participants citing (% of group)	Participant vaccinated livestock last year?		Total participants citing (% of group)	Participant vaccinated livestock last year?		Total participants citing (% of group)
		yes	no		yes	no		yes	no	
**Adverse reactions (cumulative)**		**26**	**22**	**48 (34%)**	**1**	**2**	**3 (14%)**	**5**	**7**	**12 (9%)**
	“Swelling at injection site”	14	10	24	-	-	-	-	-	-
	“Swelling at injection site and weakness or death”	2	-	2	1	-	1	-	-	-
	“Vaccination causes weakness”	4	1	5	-	-	-	-	-	-
	“Weakness and animals which are already sick and pregnant ones”	2	-	2	-	-	-	-	-	-
	“Pregnancy (adverse effect on)”	1	1	2	-	1	1	4	7	4
	“Animals may die”	3	7	10	-	1	1	1	-	1
	“Animals get fever”	-	1	1	-		-	-	-	-
	“Vaccination is harmful” / “not beneficial “	-	2	2	-	-	-	-	-	-
	**Unable to access vaccination programme (cumulative)**	**2**	**8**	**10 (7%)**	**1**	**2**	**3 (14%)**	**1**	**82**	**59%**
	Lack of awareness of vaccine drive	1	2	3	-	-	-	-	10	-
	“I may not be available at the time of the vaccination drive”	-	2	2	-	-	-	-	2-	-
	“Difficulty in restraining animals”	-	-	-	1	-	1	-	-	-
	“Vet (or AHW) was not available”	1	4	5		2	2	1	70-	1
	**Other answers (cumulative)**	**24**	**27**	**51 (36%)**	**9**	**7**	**16 (73%)**	**35**	**7**	**42 (30%)**
	“Animals are healthy so there is no need to vaccinate”	-	1	1	-	-	-	-	7-	-
	Yes, there are reasons not to vaccinate livestock	1	-	1	-	-	-	-	-	-
	No, there are no reasons not to vaccinate livestock	23	26	49	9	7	16	35		35
	**Unable to answer question (cumulative)**	**21**	**13**	**32 (22%)**	**-**	**-**	**-**	**2**	**-**	**2(1.4%)**
	“I don’t know”	11	10	21	-	-	-	2		2
	Did not answer this question	10	3	11	-	-	-	-	-	-
	**Total**	**73**	**70**	**143**	**11**	**11**	**22**	**44**	**96**	**140**

**Note:** Bold type highlights cumulative totals.

Participants were asked how often they vaccinated their animals (Table 1), 82% of participants in the KTR group reported that they vaccinated their livestock every year, however only 51% of the same participants reported that they had actually vaccinated their animals in the last 12 months. Table 7 shows reasons given by farmers of KTR region for vaccinating animals annually: 93/ 142 participants (65%) stated that they ‘did not know the reason for annual vaccination’, while 22 participants (15%) stated that ‘vaccination protected animals for one year only’. Four participants identified domestic animal movements as an ongoing risk for disease transmission, while one participant cited contact with wild species as a disease risk.

**Table 7:** Reasons for annual vaccination (KTR).

Kanha (KTR) only	Vaccinated last year?		Total participants citing reason for annual vaccination
“Why do you vaccinate your animals every year?”	yes	no	
“Disease prevention”	9	4	11
“Protection only lasts one year”	15	7	22
“There is always a risk of disease outbreak / constant illness”	1	1	2
“Buying and selling animals / animal movements”	3	1	4
“Animals go to the forest and disease risk is always present”	1	-	1
“Keeping animals healthy”	1	-	1
“New diseases”	1	-	1
“Cows have been healthy or the past 2 years so vaccination not required”	-	1	1
“Village outside of buffer zone so no free vaccine available”	-	1	1
“I was not informed” (of vaccine drive)	-	3	3
“I don’t know”	42	51	93
**Total**	**73**	**69**	**142**

**Notes:** One participant did not answer the question: “Why do you vaccinate your animals every year?”.

Bold type highlights cumulative totals.

Farmer understanding of duration of immunity is an important factor in continued engagement in immunisation programmes. The 44 participants in the KNP who reported vaccinating their animals were asked about the duration of vaccination protection, 30 responded that the duration of protection was 1 year, five that it was 6 months, one that it was 3 to 4 months; two said 1 month. Six participants did not understand the concept of duration of protection.

Contact with AHWs can, unsurprisingly, be linked to improved animal health knowledge and improved vaccine uptake. When considering the KTR group, 91 (64%) stated vets or AHWs were their main source of animal health knowledge (Table 8), however when only those animal keepers who vaccinated their livestock in the last 12 months are considered, this proportion increased to 74% (54/73). Among respondents around the KNP, 64% said that observation of symptoms and disease is the main way that they learn about animal health. Among respondents from KTR and BTR, those who consider AHWs or vets to be their main source of information are more likely to access vaccination services (odds ratio 1.83, chi square test [1, n = 165] likelihood ratio = 8.344, P = 0.004). Respondents from KNP were excluded because of the low availability of access to AHWs in their area.

**Table 8:** Sources of animal health information.

Main source of animal health information	Kanha (KTR)			Bandhavgarh (BTR)			Kaziranga (KNP)		
	Participant vaccinated livestock last year?		Total participants citing	Participant vaccinated livestock last year?		Total participants citing	Participant vaccinated livestock last year?		Total participants citing
	yes	no		yes	no		yes	no	
Animal Health Workers / vets	54	37	91	3	1	4	-	1	1
Discussion with family and community	13	26	39	2	7	9	3	14	17
Observation of symptoms	2	-	2	6	3	9	33	57	90
Don’t know	4	7	11	-	-	-	8	24	32
**Total**	**73**	**70**	**143**	**11**	**11**	**22**	**44**	**96**	**140**

**Note:** Bold type highlights cumulative totals.

It should be noted that there was a well-established network of AHWs serving the KTR group. In the BTR, AHWs were also present, but their service was still becoming established in that area. In the KNP there was no formal system of AHWs, however a network of veterinary clinicians and veterinary field assistants provide animal health services and advice.

### 3.2 KNP in-depth interviews

In-depth interviews were carried out in three villages immediately adjacent to the KNP boundary. Because of their proximity to the KNP, these villages have been well penetrated by vaccination teams for a number of years. The 17 in–depth interviews encompassed 18 households. Eight interviews were with a single main respondent: five men and three women; nine interviews were with a pair of main respondents: four married couples, two daughters with their mothers, two sons with their mothers, and one pair of brothers. One interview was with three main respondents, a married couple and a male neighbour representing a separate household. The age range of main participants was 24–65 years (mean age 40 years). Additional family members or friends frequently joined interviews. From these 18 households, 17 gave information about livestock vaccinations, 14 which reported vaccinating their cattle (82%), and three households stated that they did not vaccinate their cattle. Among those households that had been accessing vaccination services, three reported immunising livestock for one to four years; three for five to nine years; six for approximately ten years; and two for more than ten years.

“(I have vaccinated my cattle) *since 2015*, *when it was done by State Animal Husbandry Department*. *I did not know about vaccination before that*. *I will continue to do it…*”

                Interviewee 2, male 49 years.

Knowledge by farmers of the purpose and benefits of vaccination assists in engagement in immunisation programmes. Generally there was an understanding among interviewees that vaccination protects from disease, although there was frequent misunderstanding about which diseases are protected against. Considering the 14 households that report accessing vaccination services, nine identified immunisation as providing protection from FMD (“*chaboka”*); additionally two of these households also identified blackquarter (“*moh-bis-oni”*), and one HS (“*golphulla*”) these diseases are included in a widely used combined vaccine. However, five households mistakenly reported vaccination to be protective against bloat (“*pet fulla*”); three against pox (“*Bohonta*”); two against diarrhoea (“hagoni”); and one “all kinds of diseases”. Three households stated that did not know against which diseases immunisation could help to protect their animals. The interviewees stated that they benefited from frequent livestock vaccination programmes to help protect wild species within the National Park.

“*If there is an outbreak then the Forest Department come and vaccinate*. *It protects against Pet-phulla* (bloat), *Chaboka* (FMD), *Moh-bisoni* (BQ), *Bohonta* (pox), *and protects against all kinds of diseases*.*”*

                Interviewee 15, female 45 years.

Only one of the 17 interviewees said they did not vaccinate their animals due to a mistrust of vaccination:

“*No*. *I did* (vaccinate) *only once- the cow’s neck swelled*, *but she became well again*. *After that I am scared of vaccination*.*”*

                Interviewee 16, male 56.

Despite these participants being relatively well informed about the benefits of vaccination, discussions often revealed passive attitudes to accessing livestock vaccines:

“*Yes*. *I have vaccinated my animals for many years*, *I only do it if the Forest Department or some other organisation (NGO) come here to do it*. *Prevents diseases*: *Kurra patta* (FMD), *pet phulla* (bloat), *hagoni* (diarrhoea).*”*

                Interviewee 10, female 33 years

“*Yes*, (I have vaccinated) *cows and oxen for the last 10 years*. *Protects them from Chaboka (FMD) and other diseases*. *I do because Forest Department initiatives provide* (vaccination) *for free*.*”*

                Interviewee 7, male 42 years

These testimonials, while positive about the benefits of vaccination, indicate little motivation to actively access or pay for livestock immunisation. Some other farmers who were intermittently participating in immunisation drives, expressed indifference to accessing vaccination services:

“*Usually we don’t use vaccine*, *I don’t really know why*, *we just don’t*. *We only vaccinate when the government or some other organisation* (NGO) *come to the village and do it*.*”*

                Interviewee 9, male 43 years.

“*No*, (I did not vaccinate last year) *Doctor didn’t come*. *I don’t know what to give*. *I really don’t think about giving medicine to an animal which is not sick*.*”*

                Interviewee 1, male 45 years.

Some participants were very keen that their animals should be vaccinated, and demonstrated an astute attitude to accessing vaccination services. Only one participant mentioned purchasing vaccines themselves:

*“*(Vaccination) *prevents disease*. *Vaccination drives occur because of Forest Department initiatives to protect wild animals*. *I consult of Forest Department staff and the Eco Development Committee in order to take advantage of these disease prevention initiatives*. *I have seen animals being vaccinated at times for my whole life*.*”*

                Interviewee 3, female 30.

*“March to April* (before the flood) *is the best time to vaccinate animals*. . . . *If vaccination is done in the proper time Chaboka never occurs…*. *I used to consult Doctor*, *but will always vaccinate if vaccine is available*, *but because of poor availability of vaccine the Forest Department don’t always come…*. *Yes* (I have vaccinated my cattle) *since 2001*. *It prevents disease; Pet-phulla* (bloat), *Gol-phulla* (HS), *Chaboka* (FMD), *Bohonta* (Pox/ skin disease). *There are different vaccines for different diseases*. *I always keep in touch with Forest Department and use vaccine if available*. *If vaccine is not available sometimes we go to the pharmacy and buy it privately and do it ourselves*.*”*

                Interviewee 11, male 58 years

## 4. Discussion

The study revealed a positive attitude to livestock vaccination among participants in all three locations, only a minority of participants expressed mistrust of vaccination or fear of negative consequences. Participants with some knowledge of vaccination more commonly accessed vaccination services. Having access to an AHW or vet for livestock health information had a significant positive effect on vaccine uptake. Predictably, difficulty accessing vaccination services was strongly associated with not vaccinating livestock, and it is reasonable to assume a direct link between these two factors. Surprisingly, fear of adverse reactions to vaccines was not found to be significantly associated with not immunising livestock. The main reasons given by participants for not vaccinating their animals during the previous year were similar to those identified in human medicine [38]). In particular, these were not being available at the time of the vaccine drive, or not being informed of the vaccine drive in a timely fashion, hence unable to present their animals. Further factors related to the need of livestock to graze, difficulty restraining animals, and the temporary and mobile nature of veterinary vaccination drives. There were no trends observed linking the age of the participants in any group to their decisions relating to livestock vaccination, nor gender in the KNP group only. The low participation of women farmers in the KTR and BTR groups, despite being contacted by female researchers may also be indicative of poor participation in immunisation programmes also, however further work is required to determine this. Factors such as the location and timing of vaccinations, restraint of animals, and access to pre- immunisation education initiatives, may affect the ability of women livestock keepers’ ability to access immunisation programmes may be a significant barrier to achieving adequate levels of vaccination coverage as well as disadvantaging these women. Consequently, the mitigation of these factors should be central to the design of immunisation programmes. Considering the KTR group only, which was well served by AHWs, it can be seen that livestock keepers who considered AHWs to be their main source of animal health information were more likely to access vaccination services, in keeping with findings in the field of human public health [39, 40]). It is of concern that many participants in this study who had vaccinated their animals did not know against which diseases. Also of concern are the participants who believed that vaccination protected against many or all diseases, or cured disease, as these opinions could lead to disappointment and disenfranchisement from vaccination programmes.

The findings of the study are limited by the number of households and locations contacted; that the sample was random rather than stratified for tribe or caste; the veracity of participants’ responses; and seasonal variation in attitudes. The high proportion of female respondents in the KNP area is commendable, though the low female participation in the KTR and BTR indicates the need for further study to properly understand the experiences of these women livestock keepers. As livestock keepers were not asked if they identified with any tribe or caste it is not possible to assess if this has any influence on access to livestock vaccination services, and further work is required to elucidate what, if any, are the effects of tribal or caste identity on livestock immunisation uptake. Different researchers collected the data in each region. The use of slightly different methodologies by each of these researchers necessitates careful consideration of the findings when comparing the different regions. In addition, it would be very helpful to know more about the opinions of those livestock keepers in the KNP who did not vaccinate their livestock in the last year, any barriers that prevented them from accessing vaccination services, and whether they would be prepared to pay for vaccination. It would be most helpful to collect information from more animal keepers in villages immediately adjacent to the KNP that were engaged by the 2018–19 immunisation drive to better understand these farmers experiences. The sample size from the BTR was very much smaller than the other two sites, which makes direct comparison difficult, but the information gained is nevertheless valuable.

Vaccination drives may be planned, or reactive in response to a disease outbreak. Reactive vaccination leads to suboptimal vaccine usage, as single doses of vaccine are administered in the face of an outbreak, rather than one or more doses being administered prior to the anticipated period of risk. Planned vaccination programmes facilitate efficient use of staff and resources; ensure availability of sufficient vaccine; optimise the timing of administration to precede local periods of maximum disease risk; allow full primary courses to be given, with booster vaccinations in subsequent vaccination cycles; and integrate with farming cycles maximising antibody in colostrum for passive transfer of maternal immunity to neonates. Structured work programmes could ensure that villages are visited in a systematic fashion.

A reactive approach to immunisation, though it provides some measure of control over a disease outbreak [53] leads to understandable difficulties in properly publicising initiatives in advance; a major difficulty identified by participants in this study in accessing vaccination services. Immunisation programmes planned well in advance allow community input, enabling the programme to be scheduled for dates which do not to coincide with other periods of maximal agricultural activity, such as planting or harvesting of crops. The Forest Department led cattle immunisation programme on the fringe of the KNP exemplifies some of the advantages of community input and engagement in programme delivery and scheduling immunisation to precede the period of greatest risk [46]. Vaccination activities can be timed during the day to simplify the presentation of animals; and proper publicity of events enables livestock keepers to plan, and prepare to keep their animals at or close by their home on the day of immunisation. Information provided to the vaccination team by the community in advance of the day of immunisation assists the vaccinators in provisioning sufficient time, staff and vaccine stocks to fully and efficiently carry out their work.

The study found lack of knowledge of vaccination to be commonplace, even among livestock keepers who were fully engaged in immunisation programmes. Areas of poor knowledge included diseases protected from; duration of protection; which animals require vaccination; and unrealistic expectations. Many participants demonstrated little knowledge of risks leading to the spread of disease. Among the minority of participants who expressed mistrust of vaccination, there were misconceptions about potential risks posed by vaccine administration. Planned vaccination programmes should allow time for relevant educational activities targeting livestock keepers and their families. Programmes could include village meetings, discussions with AHWs, and distribution of printed materials. Farmer education could increase engagement through knowledge; dispel inaccurate and unhelpful ideas regarding vaccination; and raise awareness of disease risk reduction and basic biosecurity measures.

Few respondents in this study could be considered to be taking ownership of the responsibility for vaccination of their animals. These passive attitudes are fostered by the history of free vaccination drives, demonstrated by the response “but we don’t pay for vaccination”. Only respondents in the KNP were willing to pay for immunisation, numerous factors including education, culture, provision of human vaccination services and information, may influence this, it may also be that the scarcer provision of vaccination in this region has added value to the service. Where locally appropriate payment of a small monetary contribution by animal keepers for vaccination could be considered to encourage personal responsibility for animal health and disease prevention, in keeping with the findings of previous studies [41, 42], however such schemes should be implemented with care to prevent this resulting in decreased vaccine uptake. Such a step should only be undertaken following appropriate vaccine education. Other goals, such as protection of wild species through ring vaccination, should be carefully considered before instigating this measure.

This study shows that the drivers behind and barriers to uptake of livestock immunisation services by smallholder farmers to be complex, and in some cases regionally variable. As increased livestock vaccination is a key tool in the achievement of United Nations Sustainable Development Goals it is imperative that as well as undertaking immunisation programmes further work must be done both to better understand the challenges to effective livestock immunisation, and to educate smallholder farmers about the causes of disease and benefits of livestock immunisation.

## 5. Recommendations

Based on the findings of this study, the following recommendations regarding the planning and undertaking of vaccination programmes for smallholder livestock farmers on the fringes of natural landscapes can be considered where locally appropriate:

- Vaccination drives should be planned in advance, timed to provide protection at the period of greatest anticipated disease risk.
- The date and times of the vaccination teams work in each village should be publicised, in that village, well in advance of the event, to allow livestock keepers to prepare properly to present their animals.
- Relevant farmer education programmes should precede immunisation programmes.
- Vaccination drives must properly engage and enfranchise beneficiaries. Vaccination and education activities should be planned in a locally sensitive manner to maximise opportunities for women farmers to be involved. Local committees should be consulted and given some responsibilities for organising the programme in their own village
- Payment of a small monetary contribution by animal keepers for vaccination could be considered, where locally appropriate, in order to encourage responsibility for animal health and disease prevention.

## Supporting information

S1 File (DOCX)

S2 File (DOCX)

S3 File (XLSX)

S4 File (XLSX)

S5 File (XLSX)

## References

[1] Wiggins S., Keats S. Smallholder agriculture’s contribution to better nutrition. *African Journal of Food*, *Agriculture*, *Nutrition and Development*. June, 2013, Vol.13(3) p. S24–S57.

[2] Ricciardi V., Ramankutty N., Mehrabi Z., Jarvis L., Chookolingo B. How much of the world’s food do smallholders produce? *Global Food Security*, Volume 17, June 2018, Pages 64–72.

[3] Lalljee SV, Soundararajan C, Singh YD, Sargison ND. The potential of small ruminant farming as a means of poverty alleviation in rural southern India. *Tropical Animal Health and Production*, 2018, 51, 303–11, doi: 10.1007/s11250-018-1686-4 30112734

[4] Sargison N.D. The critical importance of planned small ruminant livestock health and production in addressing global challenges surrounding food production and poverty alleviation. *New Zealand Veterinary Journal*, 2020, Vol.68(3), pp.136–144 doi: 10.1080/00480169.2020.1719373 31968203

[5] FAO (2017) Livestock solutions for climate change. http://www.fao.org/3/I8098EN/i8098en.pdf Accessed 14/10/20.

[6] Hopker A, Pandey N, Goswami J, Hopker S, Saikia R, Jennings A, et al (2020) Colostrum provision and care of calves among smallholder farmers in the Kaziranga region of Assam, India. *PLoS ONE* 2020, doi: 10.1371/journal.pone.0228819 32160186 PMC7065800

[7] Campbell Z A; Otieno L; Shirima G M; Marsh T L; Palmer G H (2019) Drivers of vaccination preferences to protect a low-value livestock resource: Willingness to pay for Newcastle disease vaccines by smallholder households. *Vaccine*, 03 January 2019, Vol.37(1), pp.11–18 doi: 10.1016/j.vaccine.2018.11.058 30478006 PMC6290109

[8] The World Bank (2020). https://data.worldbank.org/topic/agriculture-and-rural-development?locations=IN&most_recent_value_desc=false Accessed 14/10/20.

[9] Agricultural Census of India 2016. http://www.indiaenvironmentportal.org.in/files/file/Agriculture%20Census%202015-16.pdf Accessed 14/10/20.

[10] Dhar, V. Agriculture Census in India 2020 www.fao.org/fileadmin/templates/ess/ess_test_folder/Workshops_Events/APCAS_24/PPT_after/APCAS-12-31-Agri_Census_India_APCAS24.pdf Accessed 14/10/20.

[11] Hopker A; Pandey N; Dhamorikar A; Hopker S; Gautam P; Pandey S; et al. Delivery and evaluation of participatory education for animal keepers led by veterinarians and para-veterinarians around the Kanha Tiger Reserve, Madhya Pradesh, India. *PLoS ONE*, 2020, Vol.13(8): e0200999. 10.1371/journal.pone.0200999 PMC6071983 30071034

[12] Roth J. A. Veterinary Vaccines and their Importance to Human and Animal Health. *Procedia in Vaccinology*, 2011, Vol.5, pp.127–136 doi: 10.1016/j.provac.2011.10.009 32288915 PMC7128871

[13] Ranabijuli S., Mohapatra J.K., Pandey L.K., Rout M., Sanyal A., Dash B.B., et al. ’Serological Evidence of Foot-and-Mouth Disease Virus Infection in Randomly Surveyed Goat Population of Orissa, India’ *Transboundary and Emerging Diseases*. 2010, 57, Pp. 448–454 doi: 10.1111/j.1865-1682.2010.01161.x 20723161

[14] Subramaniam S., Pattnaik B., Sanyal A., Mohapatra J. K., Pawar S. S., Sharma G. K., et al. Status of Foot‐and‐mouth Disease in India. *Transbound*. *Emerg*. *Dis*. 2013, 60, 197 doi: 10.1111/j.1865-1682.2012.01332.x 22551096

[15] Pattnaik B., Subramaniam S., Sanyal A., Mohapatra J. K., Dash B. B., Ranjan R., et al. Foot‐and‐Mouth Disease: global status and future road map for control and prevention *India*. *Agric*. *Res*. 2012, 1, 132.

[16] Hegde R; Gomes A R; Giridhar P; Kowalli S; Shivashankar B P; Sudharshana K J; et al. Epidemiology of foot and mouth disease in Karnataka state, India: a retrospective study *VirusDisease*, 2014, Vol.25 (4), p.504–509. doi: 10.1007/s13337-014-0239-3 25674631 PMC4262310

[17] Sharma G.K.; Mahajan S.; Matura R.; Biswal J.K.; Ranjan R.; Subramaniam S.; et al. Herd Immunity Against Foot and Mouth Disease under different vaccination practices in India. *Transboundary and Emerging Diseases*, August 2017, Vol.64(4), pp.1133–1147 doi: 10.1111/tbed.12478 26920973

[18] Government of India, Department of Animal Husbandry and Dairying. (Foot and mouth disease control programme (FMD-CP). 2020 http://dadf.gov.in/division/more-about-fmdcp1. Accessed 14/10/20.

[19] Mondal S. P.; Yamage M. A Retrospective Study on the Epidemiology of Anthrax, Foot and Mouth Disease, Haemorrhagic Septicaemia, Peste des Petits Ruminants and Rabies in Bangladesh, 2010–2012 *PLoS ONE*, 2014, Vol.9(8) 10.1371/journal.pone.0104435 PMC4125197 25101836

[20] OIE. Haemorrhagic septicaemia. 2013 https://www.oie.int/fileadmin/Home/eng/Animal_Health_in_the_World/docs/pdf/Disease_cards/HAEMORRHAGIC_SEPTICEMIA.pdf Accessed 14/10/20.

[21] Singh B; Shiv P; Verma M. R; Sinha D. K. Estimation of economic losses due to Haemorrhagic Septicaemia in cattle and buffaloes in India *Agricultural Economics Research Review*, 2014, Vol.27(2), pp.271–279

[22] Kumar P.; Singh V.; Agrawal R.; Singh S. Identification of Pasteurella multocida isolates of ruminant origin using polymerase chain reaction and their antibiogram study *Tropical Animal Health and Production*, 2009, Vol.41(4), pp.573–578 doi: 10.1007/s11250-008-9226-2 18759064

[23] Shivachandra S. B; Viswas K. N; Kumar A. A A review of hemorrhagic septicemia in cattle and buffalo Animal Health Research Reviews, 2011, Vol.12(1), pp.67–82 doi: 10.1017/S146625231100003X 21676341

[24] Sultana M., Ahad A., Biswas P.K, Rahman M.A. & Barua H. ’Black Quarter (BQ) Disease in Cattle and Diagnosis of BQ Septicaemia Based on Gross Lesions and Microscopic Examination’ *Bangladesh Journal of Microbiology*, 2008. Pp. 13–16

[25] Useh N.M., Nok A.J. and Esevio K.A.N. Blackleg in Ruminants. CAB Reviews *Perspectives in Agriculture Veterinary Science Nutrition and Natural Resources*, 2006, 1(040) doi: 10.1079/PAVSNNR20061040 Accessed 14/10/20.

[26] Zahid U N; Kumar S. M; Randhawa S S; Randhawa S S; Hassan M. N. Black quarter in crossbred dairy cattle—a case report. *Veterinary World*, Dec 2012, Vol.5(12), p.767(4)

[27] Niamatullah M. Economic losses due to high incidence of black quarter disease in cattle and buffaloes and its treatment in district Dera Ismail Khan.(report) *Pakistan Journal of Science*, June 30, 2011, Vol. 63(2)

[28] Ziech R. E., Gressler L.T., Frey J., & Vargas A.C. Blackleg in cattle: current understanding and future research needs. *Ciência Rural*, 2018 48(5), e20170939. Epub May 17, 2018. 10.1590/0103-8478cr20170939 Accessed 14/10/20.

[29] Reddy G. S and Srinivasan V. A. A comparative study of the serological response of Al-gel and oil- base vaccines against Black Quarter. *Indian J*. *Animal Sci*. 1997. 67(9): 764–765

[30] Siembieda J., Kock R., McCracken T., & Newman S. The role of wildlife in transboundary animal diseases. *Animal Health Research Reviews*, 2011, 12(1), 95–111. doi: 10.1017/S1466252311000041 21615975

[31] Rout M.; Subramaniam S.; Das B.; Mohapatra J.K.; Dash B.B.; Sanyal A.; et al. Foot-and-mouth disease in wildlife population of India *Indian Journal of Animal Research*, April 2017, Vol.51(2), pp.344–346

[32] Chandranaik B M; Hegde R; Shivashankar B P; Giridhar P; Muniyellappa H K; Kalge R; et al. Serotyping of foot and mouth disease virus and Pasteurella multocida from Indian gaurs (Bos gaurus), concurrently infected with foot and mouth disease and haemorrhagic septicaemia *Tropical animal health and production*, 2015–06, Vol.47 (5), p.933–937 25894817 10.1007/s11250-015-0811-x

[33] Thomson G.R; Vosloo W; Bastos A D S; Foot and mouth disease in wildlife *Virus Research* Volume 91, Issue 1, January 2003, Pages 145–161 doi: 10.1016/s0168-1702(02)00263-0 12527441

[34] Jori F; Etter E. Transmission of foot and mouth disease at the wildlife/livestock interface of the Kruger National Park, South Africa: Can the risk be mitigated? *Preventive veterinary medicine*, 2016-04-01, Vol.126, p.19–29 doi: 10.1016/j.prevetmed.2016.01.016 26848115

[35] Ward MP; Garner MG; Cowled BD Modelling foot‐and‐mouth disease transmission in a wild pig–domestic cattle ecosystem *Australian veterinary journal*, 2015–01, Vol.93 (1–2), p.4–12 doi: 10.1111/avj.12278 25622702

[36] Rathod P; Chander M; Bangar Y. Livestock vaccination in India: an analysis of theory and practice among multiple stakeholders *Revue scientifique et technique (International Office of Epizootics)*, 2016–12, Vol.35 (3), p.729–739 doi: 10.20506/rst.35.3.2564 28332655

[37] Heffernan C; Thomson K; Nielsen L. Caste, livelihoods and livestock: An exploration of the uptake of livestock vaccination adoption among poor farmers in India. *Journal of international development*, 2011–01, Vol.23 (1), p.103–118

[38] Singh S; Sahu D; Agrawal A; Vashi M D. Ensuring childhood vaccination among slums dwellers under the National Immunization Program in India—Challenges and opportunities *Preventive medicine* 2018, 112:54–60 doi: 10.1016/j.ypmed.2018.04.002 29626558

[39] Johri M; Subramanian S V; Sylvestre M-P; Dudeja S; Chandra D; Koné GK; et al. Association between maternal health literacy and child vaccination in India: a cross-sectional study *Journal of Epidemiology and Community Health* 2015, 69:849–857. doi: 10.1136/jech-2014-205436 25827469 PMC4552929

[40] Powell-Jackson T; Fabbri C; Dutt V; Tougher S; Singh K. Effect and cost-effectiveness of educating mothers about childhood DPT vaccination on immunisation uptake, knowledge, and perceptions in Uttar Pradesh, India: A randomised controlled trial. *PLoS medicine* 2008, 10.1371/journal.pmed.1002519 Accessed 14/10/20. PMC5839535 29509769

[41] Campbell Z A; Otieno L; Shirima G M; Marsh T L; Palmer G H. Drivers of vaccination preferences to protect a low-value livestock resource: Willingness to pay for Newcastle disease vaccines by smallholder households *Vaccine*, 2019-01-03, Vol.37 (1), p.11–18 doi: 10.1016/j.vaccine.2018.11.058 30478006 PMC6290109

[42] Jemberu W T; Molla W; Dagnew T; Rushton J; Hogeveen H. Farmers’ willingness to pay for foot and mouth disease vaccine in different cattle production systems in Amhara region of Ethiopia. PloS one, 2020, Vol.15 (10 October), p.e0239829 doi: 10.1371/journal.pone.0239829 33006982 PMC7531826

[43] Cox S J; Carr V B; Parida S; Hamblin P A; Prentice H; Charleston B; et al Longevity of protection in cattle following immunisation with emergency FMD A22 serotype vaccine from the UK strategic reserve *Vaccine*, 2010, Vol.28 (11), p.2318–2322 doi: 10.1016/j.vaccine.2009.12.065 20056183

[44] Reddy G. S., Antony P. X. and Srinivasan V. A. Serological response to combined vaccination of cattle against Foot-and-Mouth disease, Haemorrhagic Septicaemia and Blackquarter. *Indian J*. *Animal Sci*. 1997, 67 (7): 585–586.

[45] El Sayed E; Mossad W; Ali S; Shawky M. Studies on the duration of immunity induced in cattle after natural FMD infection and post vaccination with bivalent oil vaccine. *Veterinary World*, 2012, Vol.5 (10), p.603–608

[46] Qureshi S; Saxena H M Estimation of titers of antibody against Pasteurella multocida in cattle vaccinated with haemorrhagic septicemia alum precipitated vaccine. *Veterinary World*, 2014-04-01, Vol.7 (4), p.224–228.

[47] Shahzad W; Nasir A A; Hussain S; Mustafa N; Kausar A; Ashraf U; et al Comparative evaluation of antibody titre against p. Multocida in nili ravi buffalo calves by using hs+bq combo vaccines adjuvanted with montanide isa 50 v2 and eolane-170 *Pakistan journal of science*, 2020-12-31, Vol.72 (4), p.255

[48] Forest Department. Report on Cattle Immunisation Programme 2019 in the fringe villages of Kaziranga National Park and Tiger Reserve. Government of Assam, Eastern Assam Wildlife Division. 2019.

[49] Miller JRB, Jhala YV, Jena J, Schmitz OJ. Landscape-scale accessibility of livestock to tigers: implications of spatial grain for modeling predation risk to mitigate human–carnivore conflict. *Ecol Evol* (2015) 5: 1354–1367. doi: 10.1002/ece3.1440 25859339 PMC4377277

[50] Chouksey S; Singh S; Tomar R P; Baghel R P S; Lal S B; Bijalwan A. Human leopard conflict in Bandhavgarh Tiger Reserve: The emerging drift and community perspective. *Indian Journal of Ecology* (2017) 44(1): 58–62.

[51] Yadava M.K. Detailed report on issues and possible solutions for long term protection of the greater one horned rhinoceros in Kaziranga National Park. Government of Assam, Kaziranga National Park. 2014 https://assets.survivalinternational.org/documents/1614/park-directors-report-to-high-court-26-05-2014-01-final-a6-2.pdf Accessed 14/10/20.

[52] Tong A; Sainsbury P; Craig J Consolidated criteria for reporting qualitative research (COREQ): a 32-item checklist for interviews and focus groups *International Journal for Quality in Health Care*, 2007, Vol. 19(6), pp.349–357 doi: 10.1093/intqhc/mzm042 17872937

[53] Golde W T; Pacheco J M; Duque H; Doel T; Penfold B; Ferman G S; et al Vaccination against foot-and-mouth disease virus confers complete clinical protection in 7 days and partial protection in 4 days: Use in emergency outbreak response *Vaccine*, 2005, Vol.23 (50), p.5775–5782 doi: 10.1016/j.vaccine.2005.07.043 16153756

