# Supplementary material for: Livestock vaccination programme participation among smallholder farmers on the outskirts of National Parks and Tiger Reserves in the Indian states of Madhya Pradesh and Assam
Source: PLoS One. 2021 Aug 27;16(8):e0256684. doi: 10.1371/journal.pone.0256684 (PMC8396743; doi:10.1371/journal.pone.0256684)
Supplement: S1 File — (DOCX) [file pone.0256684.s001.docx]

# **Survey Questionnaire: Kanha and Banhavgarh Tiger Reserve regions**

Interview introduction

*“Hello, I am a student working with The University of Edinburgh. I am interested in speaking to you today about cattle disease and immunization - would you be able to spare 10 minutes and would you be happy to participate in this study?”* 🡪 VERBAL CONSENT

*If no,*

*“Thank you, have a good day.”*

*If yes,*

*“Thank you for agreeing to participate.”*

Participant details

**PARTICIPANT AGE:**

**PARTICIPANT SEX: M/F**

The interview

|  | **Question** | **Answer** |
| --- | --- | --- |
| 1 | **How many cattle and buffalo do you have in total?** |  |
| 2 (i) | **Have you heard of Foot and Mouth disease?** |  |
| (ii) | **Are your animals infected by Foot and Mouth disease, or have they been infected in the past 3 years?** |  |
| 3 (i) | **Have you heard of Black Quarter disease?** |  |
| (ii) | **Are your animals infected by Black Quarter disease, or have they been infected in the past 3 years?** |  |
| 4 (i) | **Have you heard of Haemorrhagic Septicaemia disease?** |  |
| (ii) | **Are your animals infected by Haemorrhagic Septicaemia disease, or have they been infected in the past 3 years?** |  |
| 5 | **How do you know about these diseases?** |  |
| 6 | **Which disease is the most important to you?**  **A – Foot and Mouth disease**  **B – Black Quarter**  **C – Haemorrhagic septicaemia** |  |
| 7 | **Can veterinary officials or a local livestock inspector help you to prevent disease in your cattle?** |  |
| 8 | **Did you have your animals vaccinated by injection this year?** |  |
| 9 | **Why did you vaccinate?/Why did you not vaccinate?** |  |
| 10 (i) | **Do you vaccinate your animals every year?** |  |
| (ii) | **Why do you repeat the vaccination every year?/Why don’t you repeat the vaccination every year?** |  |
| 11 | **What do you think are the benefits of vaccinating animals?** |  |
| 12 | **Do you think there are any reasons not to vaccinate your animals?** |  |
| 13 | **Do you use any other protective mechanisms against disease?** |  |
| 14 | **What do you think is the best way to protect your animals against disease?** |  |
| 15 | **If there was a cost for the vaccine, would you pay for it?** |  |
| 16 | **Can animals which you buy introduce disease to your herd?** |  |
| 17 | **Has there been less disease in cattle here since the vaccination was started?** |  |

Closing thanks

*“Thank you for your time, your input has been very valuable.”*

# **Survey Questionnaire: Kaziranga National Park region**

**Introduction**

My name is Abigail Blanton and I am a veterinary student at the University of Edinburgh in Scotland. I would like to talk to you about cattle and buffalo disease and vaccination. I will publish this information to help other people plan animal health programs in India. The questions should only take about 10 minutes of your time and your personal information will remain confidential. Would you be happy to participate?

**Participant Details**

M/F/Other:

Village:

| What animals do you have? | Cows: Buffalo: Other: |
| --- | --- |
| How many are young and how many are adults? | Young: Adults: |
| Did you vaccinate your animals this year? | Yes No I don’t know |

If they vaccinate their animals:

| Explain why you vaccinate your animals. | |  | | |
| --- | --- | --- | --- | --- |
| What are the disadvantages to vaccinating your animals, if any? | |  | | |
| How often do you vaccinate? | | Every 6 months  Once a year  Every few years  Once in the animal’s lifetime  When the person comes to do it  I don’t know | | |
| Why do you vaccinate that often? | |  | | |
| How long does the vaccine last? | | 6 months  One year  Two years  More than two years  The animal’s lifetime  I don’t know | | |
| Who administers the vaccine? | | Government veterinarian  Private veterinarian  Veterinary assistant  You (the farmer)  Other | | |
| Where do you get the vaccine from? | | Government veterinarian  Private veterinarian  Medicine shop  Other | | |
| What disease are you vaccinating for? | | Foot and Mouth Disease  Anthrax  Haemorrhagic Septicaemia  Black Quarter  Other (PPR, GP, etc)  I don’t know | | |
|  | Have you heard of this disease? | | | Have any of your animals been affected by this disease? |
| Foot and Mouth Disease |  | | |  |
| Haemorrhagic Septicaemia |  | | |  |
| Black Quarter |  | | |  |
| Anthrax |  | | |  |
| How much does it cost to vaccinate your animal? | | | Free  Less that Rs. 5  Rs. 5-30  More than Rs. 30  I don’t know | |
| How much would you pay to vaccinate your animal? | | | Nothing  Less than Rs. 5  Rs. 5-30  More than Rs. 30 | |
| What other methods of disease prevention do you use? | | |  | |
| Can animals that you buy introduce disease to your herd? | | | Yes No I don’t know | |
| Has there been less disease in cattle here since the vaccination was started? | | | Yes No I don’t know | |

If they don’t vaccinate their animals:

| Explain why you do not vaccinate your animals. | |  | | |
| --- | --- | --- | --- | --- |
| What are the advantages to vaccinating your animals, if any? | |  | | |
| Would you vaccinate if it were cheaper or free? | | Yes No I don’t know | | |
| What other methods of disease prevention do you use? | |  | | |
| Can animals that you buy introduce disease to your herd? | | Yes No I don’t know | | |
| Has there been less disease in cattle here since the vaccination was started? | | Yes No I don’t know | | |
|  | Have you heard of this disease? | | Have any of your animals been affected by this disease? |  |
| Foot and Mouth Disease |  | |  |  |
| Haemorrhagic Septicaemia |  | |  |  |
| Black Quarter |  | |  |  |
| Anthrax |  | |  |  |
